# Evaluation of novel xylose-fermenting yeast strains from Brazilian forests for hemicellulosic ethanol production from sugarcane bagasse

**DOI:** 10.1007/s13205-013-0145-1

**Published:** 2013-06-11

**Authors:** Sabrina E. Martiniano, Anuj K. Chandel, Luma C. S. R. Soares, Fernando C. Pagnocca, Sílvio S. da Silva

**Affiliations:** 1 Department of Biotechnology, Engineering School of Lorena, University of São Paulo, Estrada Municipal Do Campinho, P.O. Box 116 12.602.810, Lorena, SP Brazil; 2 Centro de Estudos de Insetos Sociais, Universidade Estadual Paulista Júlio de Mesquita Filho, P.O. Box 199, Rio Claro, SP CEP 13506-900 Brazil

**Keywords:** *Scheffersomyces shehatae*, Second-generation ethanol, Xylose, Sugarcane bagasse, Hemicellulose hydrolysate

## Abstract

Bioconversion of hemicellulosic hydrolysates into ethanol with the desired yields plays a pivotal role for the overall success of biorefineries. This paper aims to evaluate the ethanol production potential of four native strains of *Scheffersomyces shehatae* (syn. *Candida shehatae*) viz. *S. shehatae* BR6-2AI, CG8-8BY, PT1-1BASP and BR6-2AY, isolated from Brazilian forests. These strains were grown in commercial d-xylose-supplemented synthetic medium and sugarcane bagasse hemicellulose hydrolysate. *S. shehatae* BR6-2AY showed maximum ethanol production [0.48 ± 0.019 g g^−1^, 95 ± 3.78 % fermentation efficiency (FE)] followed by *S. shehatae* CG8-8BY (0.47 ± 0.016 g g^−1^, 93 ± 3.12 % FE), *S. shehatae* BR6-2AI (0.45 ± 0.01 g g^−1^, 89 ± 1.71 % FE) and *S. shehatae* PT1-1BASP (0.44 ± 0.02 g g^−1^, 86 ± 3.37 % FE) when grown in synthetic medium. During the fermentation of hemicellulose hydrolysates, *S. shehatae* CG8-8BY and *S. shehatae* BR6-2AY showed ethanol production (0.30 ± 0.05 g g^−1^, 58 ± 0.02 % FE) and (0.21 ± 0.01 g g^−1^, 40 ± 1.93 % FE), respectively.

## Introduction

The demand for alternative and sustainable fuel source has been raised in the last few years due to diminishing petroleum resources, regular price hikes of gasoline and environmental pollution. Ethanol derived from renewable biomass has shown promising results for replacing partially or totally gasoline (Goldemberg 2007). Bioethanol can be produced directly by fermentation of sugars from sugarcane, sugar beet and corn (first generation ethanol) or vegetal biomass such as crop residues, forestry waste and kitchen waste (second-generation ethanol) (Lin and Tanaka 2006). Among the crop residues, sugarcane bagasse (SB) is generated in foreseeable amount in countries like Brazil, India, China and Australia and could be a promising feedstock for biorefineries (Chandel et al. 2012).

Dilute sulfuric acid-mediated pretreatment effectively solubilizes the hemicellulosic fraction of SB into simple sugars and thus ameliorates the accessibility of cellulose to cellulolytic enzymes. Bioconversion of hemicellulosic sugars into ethanol with satisfactory yields is essential for the total ethanol production from lignocellulosic materials (Saha 2003). Dilute acid hydrolysis leads to the generation of some undesired products such as furfural, 5-hydroxymethylfurfural (HMF), weak acid, extractives and phenolic compounds (Chandel et al. 2007; Milessi et al. 2012). These compounds are toxic to the microorganisms and are required to be removed from hydrolysates to obtain satisfactory ethanol yields during microbial fermentation (Canilha et al. 2013).

The ideal microorganism for the production of ethanol would be the one that can equally convert pentose and hexose sugars into ethanol. The best-known alcohol-fermenting organisms, *Saccharomyces cerevisiae* and *Zymomonas mobilis*, are capable of fermenting only hexose sugars and sucrose into ethanol. However, pentose-fermenting organisms are limited including *Pichia* (*Scheffersomyces*) *stipitis*, *S. shehatae* and *Pachysolen tannophilus* (Saha 2003). Among the d-xylose-fermenting microorganisms, *Scheffersomyces shehatae* syn. *Candida shehatae* (Urbina and Blackwell 2012) is one of the most studied and has shown promising ethanol production from a variety of raw materials (du Preez 1994; Abbi et al. 1996; Chandel et al. 2007). This microorganism is capable of metabolizing d-xylose as well as glucose and presents high tolerance to ethanol (du Preez 1994). Bioprospecting is useful for finding new microbial strains from natural or industrial habitats with specific properties. d-xylose-metabolizing microorganisms have been isolated from fruits, insect frass, tree exudates and insect intestines (Ferreira et al. 2011). The four *S. shehatae* yeast strains (*S. shehatae* BR6-2AI, CG8-8BY, PT1-1BASP and BR6-2AY) used in the present study were isolated from different natural habitats.

The present study is the first approach to evaluate the fermentative potential of these novel strains of *S. shehatae* for second-generation ethanol production from sugarcane hemicellulosic hydrolysate and d-xylose-supplemented fermentation medium.

## Materials and methods

### Sugarcane bagasse and preparation of hemicellulosic hydrolysate

Sugarcane bagasse was provided by Usina Santa Fé at Nova Europa/São Paulo, Brazil. It was acid hydrolyzed by 100 mg H_2_SO_4_/g of dry bagasse at 1:10 of solid/liquid ratio, 121 °C for 10 min in a hydrolysis reactor of 100 l capacity (Milessi et al. 2012). This reactor is made up of stainless steel (SS 316) and located at the Department of Biotechnology, Engineering School of Lorena (EEL)-USP, Lorena, Brazil.

After the hydrolysis, hemicellulosic hydrolysate was recovered and subsequently concentrated in a vacuum evaporator of 30 l at 70 °C until xylose concentration reached about 60 g l^−1^ followed by filtration and detoxification as shown by Milessi et al. (2012). The vacuum concentrator was also indigenously fabricated and located at the Department of Biotechnology, Engineering School of Lorena (EEL)-USP, Lorena, Brazil. This detoxification procedure consisted of raising the pH of the hydrolysate by adding calcium oxide to pH 7.0, followed by pH reduction to 5.5 with phosphoric acid (85 % of purity). Activated charcoal 2.5 % (w/v) was then added in neutralized hydrolysate and incubated at 30 °C, 200 rpm for 60 min (Alves et al. 1998). Thereafter, the hydrolysate was vacuum filtered by Whatman filter paper for the removal of precipitates. The detoxified hydrolysate was autoclaved at 0.5 atm (110 °C) for 15 min and used for subsequent fermentation assays.

### Microorganism and inoculum preparation

Four strains of *S. shehatae*: BR6-2AI, CG8-8BY, PT1-1BASP and BR6-2AY were kindly provided by the Centre of Microbial Resources, UNESP, Rio Claro, Brazil. *S. shehatae* BR6-2AI and *S. shehatae* BR6-2AY were isolated from bromeliads. *S. shehatae* CG8-8BY and *S. shehatae* PT1-1BASP were isolated from mushroom and *Euterpe* sp., respectively. Stock cultures were maintained on YPMG agar (0.3 % yeast extract, 0.5 % peptone, 0.3 % malt extract, 1.0 % glucose and 2.0 % agar) at 4 °C.

For inoculum preparation, loopful cultures were transferred to 250 ml Erlenmeyer flasks containing 100 ml of YPX medium (10.0 g yeast extract l^−1^, 20.0 g peptone l^−1^, 30.0 g xylose l^−1^, pH 6.0). The flasks were incubated at 30 °C, 200 rpm for 24 h. After 24 h of incubation, the cells were recovered by centrifugation (2,000×*g*, 20 min) at room temperature, washed, centrifuged again and suspended in sterile distilled water to obtain an initial concentration of 0.5 g l^−1^.

### Fermentation medium and conditions

Fermentative performance of four *S. shehatae* strains was determined in synthetic medium (YPX medium) containing 50 g xylose l^−1^. Fermentation assays were performed in 250 ml Erlenmeyer flasks containing 100 ml of YPX medium, inoculated with 0.5 g cells l^−1^, at 30 °C, 200 rpm for 48 h. The strains which showed better ethanol yields in synthetic media (CG8-8BY and BR6-2AY) were employed for the fermentation of detoxified sugarcane bagasse hydrolysate supplemented with 3 g yeast extract l^−1^. Erlenmeyer flasks (250 ml) containing 100 ml of medium were incubated at 30 °C, pH 5.0, 150 rpm for 96 h. Fermentation runs were monitored through periodic sampling to determine the cell growth, sugar consumption and ethanol production.

### Analytical methods and determination of fermentation parameters

Hydrolysate samples were filtered in Sep-Pak C18 and analyzed for the estimation of xylose, glucose, arabinose, acetic acid, xylitol and ethanol concentrations by high-performance liquid chromatography (HPLC, Agilent Technology, USA). Chromatograph (A1100 EUA) equipped with column Bio-Rad AMINEX HPX-87H (300 × 7.8 mm) was used at 45 °C, 20 μl of flow rate, with refractive index detector, 0.01 N sulfuric acid as eluent and a flow rate of 0.6 ml/min. Furfural and HMF concentration was also estimated by HPLC (Waters 2487, USA) equipped with column HP-RP 18 (200 × 4.6 mm) at 25 °C, 20 μl flow rate, ultraviolet detector SPD-10A UV–VIS (276 nm), eluting with acetonitrile/water (1:8) with 1 % acetic acid and a flow rate of 0.8 ml/min, column temperature 25 °C and injected sample volume of 20 μl. The samples were filtered by Minisart 0.22 membranes (Sartorius AG, Goettingen, Germany) (Canilha et al. 2005; Chandel et al. 2007; Milessi et al. 2012).

During the fermentation of synthetic hydrolysates, samples were withdrawn after 0, 12, 24 and 48 h of incubation. On the other hand, samples were withdrawn after 0, 12, 24, 48, 72 and 96 h of incubation during the fermentation of sugarcane bagasse hemicellulosic hydrolysates. Cell growth was estimated by measuring the absorbance of fermentation broth at 600 nm, which was correlated to a calibration curve (dry weight vs. optical density). Ethanol yield (Y_P/S_, g g^−1^) was calculated by the ratio of ethanol concentration (g l^−1^) and substrate (glucose and xylose) consumed (g l^−1^); the ethanol volumetric productivity (*Q*_P_) was determined by ethanol concentration per time (g l^−1^ h^−1^). The fermentation efficiency (*η*%) was measured by the ratio of the yield factor obtained experimentally and the theoretical yield factor. All the fermentation experiments were carried out in triplicate, and the experimental results represent the mean of three identical sets of reactions/fermentations.

## Results and discussion

### Sugarcane bagasse hemicellulose hydrolysis

Dilute sulfuric acid hydrolytically acts on hemicellulose and converts it into sugar monomers in addition to other ingredients. The hemicellulosic hydrolysate, recovered after dilute acid hydrolysis, presented a total sugar (xylose, arabinose and glucose) concentration of 18.14 g l^−1^. Table 1 shows the compositional profile of hemicellulose hydrolysate. Xylose (16.0 g l^−1^) was the main component in hemicellulosic hydrolysate followed by arabinose (1.15 g l^−1^) and acetic acid (1.05 g l^−1^). Dilute acid hydrolysis is an effective method for the solubilization of hemicellulose into its monomeric constituents (Saha 2003). Recently, Milessi et al. (2012) reported 12.45 g l^−1^ of xylose and 0.67 g l^−1^ of glucose along with inhibitors in the hemicellulosic hydrolysate of SB under similar conditions. Earlier, Chandel et al. (2007) obtained 30.29 g l^−1^ total reducing sugars along with 1.89 g l^−1^ furans, 2.75 g l^−1^ total phenolics and 5.45 g l^−1^ acetic acid in the sugarcane bagasse acid hydrolysate. Dilute sulfuric acid-mediated thermochemical reactions at high temperatures (120–180 °C) for few minutes of residence time facilitate the cleavage of β-1, 4 xylosidic linkages in hemicellulose of SB into xylose and other by-products, leaving cellulose and lignin together but in fragile form for the precise enzymatic action (Canilha et al. 2013). The extent of action and hemicellulose solubilization during dilute sulfuric acid hydrolysis depends on the nature/type of raw material, solid to liquid ratio, temperature and the acid concentration. For instance, Mussato and Roberto (2004) obtained hemicellulosic hydrolysate of rice straw which showed 16.4 g xylose l^−1^, in conjunction with glucose (3.7 g l^−1^) and arabinose (2.6 g l^−1^). Canilha et al. (2005) observed 18.11 g l^−1^ of xylose in addition to other by-products (7.6 g glucose l^−1^ and 2.23 g arabinose l^−1^). These results show the distinctiveness of the chemical composition of acid hydrolysate due to the difference in hemicellulose composition of each vegetal species and the acid hydrolysis conditions employed (Table 1).

**Table 1:** Concentration of sugars and inhibitors in native, concentrated and detoxified sugarcane bagasse hemicellulosic hydrolysate

Compounds		Concentration (g l^−1^)		
		Native hydrolysate	Concentrated hydrolysate^a^	Detoxified hydrolysate
Sugars (g l^−1^)	Xylose	16.0	81.44	52.0
	Glucose	0.99	6.62	3.63
	Arabinose	1.15	5.77	3.00
Inhibitors (g l^−1^)	Acetic acid	1.05	2.92	1.35
	Furfural	0.42	7.89	0.001
	HMF	0.02	3.53	0.0001
pH		1.26	0.71	5.02

^a^Hemicellulosic hydrolysate concentrated fivefold from its original volume by vacuum evaporation

**Table 2:** Ethanol yield [*Y*_P/S_ (g g^−1^)], ethanol productivity [*Q*_P_ (g l^−1^ h^−1^)], fermentation efficiency [*η* (%)], xylose consumption (%), cell concentration (g l^−1^), ethanol concentration (g l^−1^) and xylitol concentration (g l^−1^) for fermentation assays by *Scheffersomyces shehatae* strains in the synthetic medium

Kinetic parameters	*S. shehatae* BR6-2AI	*S. shehatae* CG8-8BY	*S. shehatae* PT1-1BASP	*S. shehatae* BR6-2AY
*Y*_P/S_ (g g^−1^)^a^	0.45 ± 0.01	0.47 ± 0.016	0.44 ± 0.02	0.48 ± 0.019
*Q*_P_ (g l^−1^ h^−1^)^b^	0.35 ± 0.01	0.37 ± 0.009	0.36 ± 0.01	0.37 ± 0.015
*η* (%)^c^	89 ± 1.71	93 ± 3.12	86 ± 3.37	95 ± 3.78
Xylose consumption (%)^d^	99 ± 0.17	99 ± 0.14	98 ± 0.07	99 ± 0.13
Cell concentration (g l^−1^)	3.76 ± 0.162	3.72 ± 0.227	3.80 ± 0.069	3.40 ± 0.267
Ethanol concentration (g l^−1^)	17.90 ± 0.266	18.87 ± 0.156	17.27 ± 0.269	19.32 ± 0.297
Xylitol concentration (g l^−1^)	–	–	1.17 ± 0.134	–
Fermentation time (h)^e^	48	48	48	48

^a^*Y*_P/S_ (g g^−1^): correlation between ethanol (Δ*P*_ethanol_) produced and xylose (Δ*S*_xylose_) consumed

^b^*Q*_P_ (g l^−1^ h^−1^): ratio of ethanol concentration (g l^−1^) and fermentation time (h)

^c^*η* (%): percentage of the maximum theoretical ethanol yield (0.51 g ethanol/g xylose)

^d^Xylose consumption (%): percentage of initial xylose consumed

^e^Time which show the maximum ethanol production (g l^−1^) value

Dilute acid hydrolysis of lignocellulosic materials also generates toxic compounds such as furfural, 5-HMF, phenolics, weak acids and others, which negatively interfere in the fermentation process (Chandel et al. 2013). The hydrolysate was concentrated by vacuum evaporation at 70 °C to increase the sugar concentration in the solution. During vacuum evaporation, the concentration of inhibitors also increased along with the concentration of sugars. Interestingly, furfural and HMF concentrations were reduced after concentration of hydrolysate, possibly due to their volatility. Among the inhibitory compounds, acetic acid and phenolics are considered greatest growth inhibitors of microorganisms. Their presence in the fermentation medium directly influences the ethanol production performance of yeasts (Chandel et al. 2007). Acetic acid, which is mainly released during the acid hydrolysis of acetyl groups presented in xylans (du Preez 1994; Saha 2003), presents an inhibitory effect to the growth of ethanol-producing microorganisms.

Table 1 shows the hydrolysate profile after concentration and detoxification by sequential conditioning (calcium oxide-mediated neutralization and activated charcoal pretreatment). Detoxification of lignocellulose hydrolysate also caused a sugar loss despite the significant elimination of inhibitors. Almost 13 % loss in xylose concentration was observed after detoxification of concentrated hydrolysate. Our results are in close agreement with the previous study of Canilha et al. (2005), who found 14 and 21 % loss in sugars and acetic acid, respectively, after the detoxification. Acetic acid loss was slightly lower than that observed by Carvalho et al. (2005) under similar experimental conditions for sugarcane bagasse hydrolysate detoxification. The pH of the native hydrolysate was 1.25, which was reduced after vacuum concentration (0.71). After detoxification of hydrolysate, the final pH of the hydrolysate was 5.02. The process of hydrolysis with sulfuric acid and the presence of acetic acid in the hydrolysate increased the concentration of H^+^ ions in the hemicellulosic sugar solution (Saha 2003).

### Fermentation assays

#### Synthetic medium supplemented with commercial xylose

**Fig. 1:**
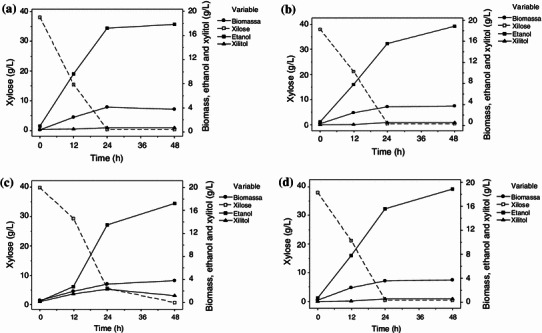
Xylose, ethanol, xylitol and biomass concentration profile during the fermentation assays of *Scheffersomyces shehatae* BR6-2AI (**a**), *S. shehatae* CG8-8BY (**b**), *S. shehatae* PT1-1BASP (**c**) and *S. shehatae* BR6-2AY (**d**) in synthetic medium (200 rpm, 30 °C and 48 h incubation). Biomass *filled circle*; xylose *opensquare box*; ethanol *filled square box*; xylitol *filled triangle*

The fermentative performance of the isolated four native yeast strains of *S. shehatae* (BR6-2AI, CG8-8BY, PT1-1BASP and BR6-2AY) was evaluated in synthetic media. Figure 1a, b, c, d shows the fermentation profile of all four strains utilizing xylose as carbon source. It is clearly evident in Fig. 1 that the maximum ethanol production by all four strains was obtained 48 h after the complete exhaustion of xylose from the fermentation medium. In all fermentation cycles, almost 90 % of xylose was consumed by the strains within 24 h, showing that xylose was the preferred choice as a main constituent of growth. Yeasts *S. shehatae* BR6-2AY, *S. shehatae* CG8-8BY, BR6-2AI and PT1-1BASP showed ethanol production of 19.32, 18.87, 17.90 and 17.27 g l^−1^, respectively. Biomass growth concomitantly increased with ethanol production. The elevated biomass production may be due to high agitation speed (200 rpm), which allows higher oxygen supply to the microorganisms, ameliorating the cellular growth. Xylitol, a by-product of the fermentation process, was produced only by *S. shehatae* PT1-1BASP and decreased after 24 h. It is associated with biomass growth, indicating that the yeast may have used the compound as carbon source.

Ethanol yield and productivity also depend on the feeding strategies of carbon source and other cultivation conditions. Similarly, du Preez et al. (1986) obtained an ethanol yield of 0.37 g g^−1^ from *S. shehatae* CSIR-Y492 in a batch bioreactor containing 50 g xylose l^−1^. Abbi et al. (1996) reported an ethanol yield of 0.43 g g^−1^ and productivity of 0.28 g l^−1^ h^−1^ from *S. shehatae* NCL-3501 utilizing 50 g xylose l^−1^) supplemented medium. According to results, yeast strains of *S. shehatae* CG8-8BY and *S. shehatae* BR6-2AY showed better ethanol production (Table 2). On account of their ethanol production profile, these strains were selected further to ferment the sugarcane bagasse hemicellulosic hydrolysate.

#### Fermentation of sugarcane bagasse hemicellulosic hydrolysate

**Fig. 2:**
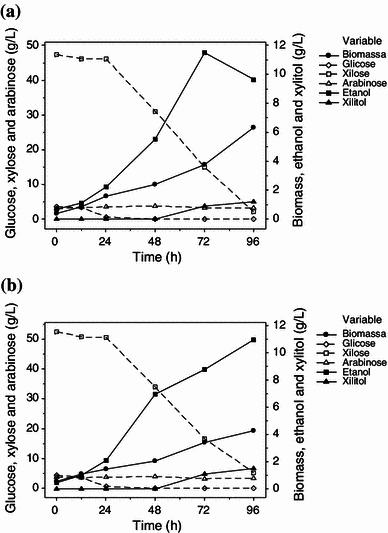
Sugars, ethanol, xylitol and biomass concentration for the fermentation assays of *Scheffersomyces shehatae* CG8-8BY (**a**) and *S. shehatae* BR6-2AY (**b**) in sugarcane bagasse hemicellulosic hydrolysate (200 rpm, 30 °C and 96 h incubation). Biomass *filled circle*; glucose *diamond*; xylose *opensquare box*; arabinose *open triangle*; ethanol *filled square box*; xylitol *filled triangle*

Among all the four yeast strains (BR6-2AI, PT1-1BASP, CG8-8BY and BR6-2AY) grown in synthetic medium, two strains (CG8-8BY and BR6-2AY) were selected for ethanol production from sugarcane bagasse hemicellulosic hydrolysate due to their improved ethanol production yields in synthetic media. The fermentation performances of *S. shehatae* CG8-8BY and *S. shehatae* BR6-2AY were assessed in sugarcane bagasse hemicellulosic hydrolysate containing 50 g xylose l^−1^ approximately and 3 g l^−1^ of yeast extract. Figure 2 shows the fermentation kinetics of both strains growing on hemicellulosic sugar solution. The total incubation time for both strains was 96 h, which is more than that of the synthetic medium. The increased incubation period is due to the presence of undesired toxic compounds in acid hydrolysates even after detoxification. *S. shehatae* CG8-8BY showed maximum ethanol production (11.49 g l^−1^) after 72 h. On the other hand, *S. shehatae* BR6-2AY exhibited maximum ethanol production (10.96 g l^−1^) after 96 h (Fig. 2).

Both the strains showed xylitol production after 72 h of incubation (Table 3). Xylitol is produced due to the necessity of cofactor regeneration in order to maintain the cellular redox balance (Kuyper et al. 2004). When xylose-reductase binds to NADPH, excess NADH may be removed forming xylitol (Kuyper et al. 2004). Xylitol accumulation is favored in micro-aeration conditions. Due to the hikes in cellular biomass, the oxygen availability in the medium is reduced (du Preez et al. 1986), affecting ethanol and xylitol production (du Preez 1994). Both the strains did not show consumption of arabinose.

**Table 3:** Ethanol yield [*Y*_P/S_ (g g^−1^)], ethanol productivity [*Q*_P_ (g l^−1^ h^−1^)], fermentation efficiency [*η* (%)], xylose consumption (%), cell concentration (g l^−1^), ethanol concentration (g l^−1^) and xylitol concentration (g l^−1^) in fermentation assays of *S. shehatae* strains in sugarcane bagasse hemicellulosic hydrolysate

Kinetic parameters	*S. shehatae* CG8-8BY	*S. shehatae* BR6-2AY
*Y*_P/S_ (g g^−1^)^a^	0.30 ± 8.63^−5^	0.21 ± 0.01
*Q*_P_ (g l^−1^ h^−1^)^b^	0.15 ± 0.005	0.11 ± 0.004
*η* (%)^c^	58 ± 0.02	40 ± 1.93
Xylose consumption (%)^d^	68 ± 1.73	90 ± 0.36
Cell concentration (g l^−1^)	3.77 ± 0.167	4.24 ± 0.516
Ethanol concentration (g l^−1^)	11.49 ± 0.339	10.96 ± 0.362
Xylitol concentration (g l^−1^)	1.0 ± 0.031	1.46 ± 0.129
Fermentation time (h)^e^	72	96

^a^*Y*_P/S_ (g g^−1^): correlation between ethanol (Δ*P*_ethanol_) produced and xylose and glucose (Δ*S*_sugars_) consumed

^b^*Q*_P_ (g l^−1^ h^−1^): ratio of ethanol concentration (g l^−1^) and fermentation time (h)

^c^*η* (%): percentage of the maximum theoretical ethanol yield (0.51 g ethanol/g xylose and glucose)

^d^Xylose consumption (%): percentage of initial xylose consumed

^e^Time which show the maximum ethanol production (g l^−1^) value

Both the strains showed preferable consumption of glucose followed by xylose. This can be related to the fact that the transport mechanism of pentose sugar assimilation can only be activated when glucose concentration in the media is exhausted (Hou 2012). The enzymatic activity of xylose-reductase and xylitol-dehydrogenase, induced by the presence of xylose and xylitol, respectively, can be repressed by glucose (Hou 2012). However, Souto-Maior et al. (2009) observed that a lower concentration of glucose stimulated the consumption of xylose due to increased activity of the glycolytic pathway in genetically modified *S. cerevisiae.*

*S. shehatae* CG8-8BY showed xylose consumption of 68 and 90 % after 72 and 96 h incubation time, respectively. There was a concomitant decrease in ethanol production and increase in cellular biomass after 72 h. *S. shehatae* CG8-8BY and *shehatae* BR6-2AY showed *Y*_P/S_ and *Q*_P_ (0.30 g g^−1^ and 0.15 g l^−1^ h^−1^) and (0.21 g g^−1^ and 0.11 g l^−1^ h^−1^), respectively (Table 3). During the xylose fermentation by yeasts, the continuous increase in cell mass even after the exhaustion of sugars is a common feature. In this condition, yeasts grow on alcohol as a carbon source, eventually reducing the total ethanol amount in the vessel (Abbi et al. 1996). Similar patterns of biomass growth were observed by Chandel et al. (2007), who reported a regular increase in the biomass of *S. shehatae* NCIM 3501 after the exhaustion of xylose in 24 h, with the utilization of ethanol as a carbon source for metabolic growth. In the present study, *S. shehatae* CG8-8BY also showed a concomitant decrease in ethanol production and increase in cellular biomass after 72 h. This yeast strain showed higher ethanol yield and productivity than a new pentose-fermenting yeast strain, *S. stipitis* UFMG-IMH 43.2, isolated from the Brazilian forest which showed ethanol production (0.19 g g^−1^ yield and 0.13 g l^−1^h^−1^ productivity) utilizing sugarcane bagasse hemicellulose hydrolysate (Ferreira et al. 2011).

Shupe and Liu (2012) evaluated the performance of two yeast strains of *S. shehatae* using sugar maple hemicellulose hydrolysate (36 g xylose l^−1^) and obtained 8.87 and 6.06 g l^−1^ of ethanol after 4 and 7 days of fermentation, respectively. Abbi et al. (1996) obtained superior ethanol yields (0.37 and 0.47 g g^−1^) from *S. shehatae* NCL-3501 utilizing rice straw hemicellulosic hydrolysate. However, Sun and Tao (2010) found ethanol concentration (16 g l^−1^, *Y*_P/S_ of 0.32 g g^−1^, *Q*_P_ of 0.19 g l^−1^ h^−1^) from *S. shehatae* CICC 1766 utilizing rice straw hemicellulosic hydrolysate. Chandel et al. (2007) found ethanol yield (*Y*_P/S_, 0.30 g g^−1^) and productivity (*Q*_P_, 0.21 g l^−1^ h^−1^) from *S. shehatae* NCIM 3501 using sugarcane hemicellulosic hydrolysate detoxified by calcium hydroxide overliming.

One of the major inhibitors in the hemicellulosic hydrolysates is acetic acid (du Preez 1994). However, in the current study, both the yeast strains were capable of metabolizing acetic acid present in the fermentation medium. Tolerance of yeasts to acetic acid is an important feature for the desired ethanol yields from lignocellulose hydrolysates. Even after detoxification of lignocellulose hydrolysates, acetic acid is present in considerable concentration. Acetic acid causes adverse effect on yeast growth due to the undissociated molecular form, which is pH dependent (Palmqvist and Hahn-Hägerdal 2000). Delgenes et al. (1996) observed the capacity of *S. shehatae* ATCC 22984 to assimilate significant quantities of acetic acid from the semi-synthetic media containing 20 g xylose l^−1^ as carbon source. Likewise, Sun and Tao (2010) verified the tolerance of this strain when grown in culture medium containing 50 g xylose l^−1^ and 1.0 g acetic acid l^−1^ and obtained 11.9 g ethanol l^−1^.

## Conclusions

Xylose is the main sugar in hemicellulosic hydrolysate of sugarcane bagasse. Microbial fermentation of hemicellulose hydrolysate with utmost ethanol yields is an important feature for economic second-generation ethanol production. There are few microorganisms capable of fermenting xylose with satisfactory yields in the presence of inhibitory compounds. Therefore, the use of new microbial strains which can produce ethanol from hemicellulosic sugar solution will essentially contribute to the success of biorefinery. *S. shehatae* strains evaluated in this study showed a great potential to ferment xylose present in the hemicellulosic hydrolysate of SB into ethanol, especially *S. shehatae* CG8-8BY (11.49 g l^−1^, yield 0.30 g g^−1^ ethanol yield). In this line, these results are promising for biorefinery development on large scale from sugarcane bagasse.
